# The Spectrum of Scarring in Craniofacial Wound Repair

**DOI:** 10.3389/fphys.2019.00322

**Published:** 2019-03-29

**Authors:** Heather E. desJardins-Park, Shamik Mascharak, Malini S. Chinta, Derrick C. Wan, Michael T. Longaker

**Affiliations:** ^1^Hagey Laboratory for Pediatric Regenerative Medicine, Department of Surgery, Stanford University School of Medicine, Stanford, CA, United States; ^2^Institute for Stem Cell Biology and Regenerative Medicine, Stanford University School of Medicine, Stanford, CA, United States

**Keywords:** wound healing, wound repair, oral mucosa, scarring, fibrosis, fibroblasts, craniofacial tissues, tissue regeneration

## Abstract

Fibrosis is intimately linked to wound healing and is one of the largest causes of wound-related morbidity. While scar formation is the normal and inevitable outcome of adult mammalian cutaneous wound healing, scarring varies widely between different anatomical sites. The spectrum of craniofacial wound healing spans a particularly diverse range of outcomes. While most craniofacial wounds heal by scarring, which can be functionally and aesthetically devastating, healing of the oral mucosa represents a rare example of nearly scarless postnatal healing in humans. In this review, we describe the typical wound healing process in both skin and the oral cavity. We present clinical correlates and current therapies and discuss the current state of research into mechanisms of scarless healing, toward the ultimate goal of achieving scarless adult skin healing.

## Introduction

Wound healing is a complex molecular process whose fundamental steps are conserved among all organ systems in the human body. The typical outcome of soft tissue defect repair in any organ is fibrosis, resulting from deposition of large amounts of abnormally organized connective tissue (Gurtner et al., 2008). The impact of fibrosis can hardly be understated; it is estimated that 45% of all deaths in the United States are attributable to fibrosis (Wynn, 2004). In humans, fibrotic tissue may develop as the result of any injury stimulus (burns, surgery, infarction, etc.) and is a major cause of wound related morbidity for millions of patients worldwide (Sen et al., 2009). In particular, dermal wound healing inevitably results in the formation of scar tissue. Skin scarring poses substantial functional and aesthetic consequences for patients, and significant scarring is common and especially detrimental in the setting of craniofacial wound repair.

While fibrosis is the most common pathway for healing in the mammalian body, wound outcomes vary widely in different healing contexts. These outcomes can range from pathological healing with exuberant fibrosis, as is seen in keloids and hypertrophic scars, to completely scarless healing, in which native tissue is perfectly regenerated. Examples across this entire spectrum can be found in craniofacial wound healing. The present review will discuss wound healing and fibrosis in the craniofacial setting, with particular emphasis on comparing skin healing (which occurs via scar formation) to healing of the oral mucosa (which is nearly scarless). We will explore recent efforts to elucidate the mechanisms underlying minimally scarring oral wound healing, which have important implications for our understanding and treatment of scarring.

## Overview of the Wound Healing Process

The ability to repair and replace damaged soft tissue is critical to the body’s ability to respond to injury. While key differences in wound healing exist between different anatomical sites, stages of development, and species, the fundamental steps of the typical adult wound healing process are conserved among mammals and are even shared between different organ systems. The basic process of adult mammalian wound healing has been extensively elucidated. Wound repair represents a tightly controlled sequence of events involving a complex network of cell types and molecular signaling pathways. Deviation from this typical sequence can lead to dysfunctional wound repair seen in humans, including a pathologic fibrotic response or chronic/non-healing wounds.

**FIGURE 1:**
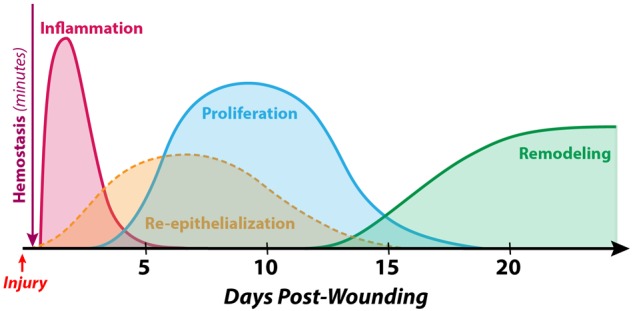
Timeline of normal human cutaneous wound healing. Wound healing following hemostasis takes place in three overlapping stages: inflammation, proliferation, and maturation/remodeling. While differences exist in the soft tissue defect repair process between different species, developmental timepoints, and anatomical sites, the fundamental steps are conserved in the vast majority of examples of adult mammalian wound healing. During the ***inflammatory phase*** (which peaks at 24–48 h post-wounding and lasts for several days), immune cells such as neutrophils and macrophages debride the wound, eliminate contaminating microbes, and secrete an array of cytokines and growth factors to recruit other cells involved in healing to the wound site. The process of ***re-epithelialization*** begins within hours of injury and accomplishes wound closure over the course of days to weeks by reestablishing a functional epithelial cell barrier. The ***proliferative phase*** (which begins 4–5 days after wounding and may last for several weeks) involves the formation of granulation tissue (by fibroblasts, endothelial cells, etc.) as a temporary substrate to fill the soft tissue defect. Finally, during the ***maturation/remodeling phase*** (the longest stage, beginning at approximately week 3 post-wounding and lasting for as long as 1–2 years), the wound bed becomes less cellular via apoptosis, and the extracellular matrix is remodeled to gradually increase in strength.

Wound healing is well understood to occur in three distinct but spatiotemporally overlapping stages: inflammation, proliferation, and maturation/remodeling (Gurtner et al., 2008) (Figure 1). While the specifics of each of these processes may vary within different wound contexts, the fundamental processes, signaling pathways, and cell types involved are stereotyped components of the mammalian response to tissue injury. Each stage is discussed in further detail below.

### Hemostasis

Tissue repair mechanisms are initially triggered by damage to the blood vessel endothelium revealing the subendothelial extracellular matrix (ECM) (Golebiewska and Poole, 2015). Exposure of matrix components such as collagen signals circulating platelets to become activated and initiate the hemostatic cascade. Local blood vessels constrict and platelets adhere to form a platelet plug which is then reinforced by fibrin polymerization, forming a clot at the injury site. The fibrin clot serves as a temporary ECM scaffold for cells involved in the early stages of repair to migrate into the wound site and is also a reservoir of important growth factors (Singer and Clark, 1999; Ghatak et al., 2015).

### Inflammation

The early inflammatory phase involves immune cell-mediated removal of pathogens, damaged cells and tissue, and other debris from the wound before new tissue can be deposited. The initial platelet adhesion and degranulation activate a cascade of inflammatory cytokines that attract immune and other cells to the wound site. These cytokines also increase vessel permeability, causing transudate leakage from capillaries which produces the classical gross manifestations of inflammation (redness, swelling, and warmth) (Velnar et al., 2009).

Neutrophils are the first inflammatory cells to arrive and serve to cleanse and debride the wound bed. Proteases secreted by neutrophils, including matrix metalloproteinases (MMPs), help to eliminate contaminating microbes from the wound site and break down ECM components to debride the wound of damaged tissue and facilitate cell migration into the wound site (Wilgus et al., 2013). Neutrophils also secrete cytokines that recruit additional inflammatory cells (e.g., monocytes) and endothelial cells and stimulate the proliferation of fibroblasts and keratinocytes to initiate re-epithelialization (Wilgus et al., 2013). Once the wound is cleared of microbes, neutrophils are typically eliminated from the wound via extrusion, apoptosis, and phagocytosis (Velnar et al., 2009). Notably, neutrophils may persist abnormally in the chronic wound setting, and continued protease production can cause sustained tissue damage and impaired healing (Wilgus et al., 2013).

Monocytes are the next inflammatory cells to enter the wound site, arriving 48 to 72 h following injury (Velnar et al., 2009). Monocytes are attracted to the wound by products of ECM breakdown and molecular factors such as platelet-derived growth factor (PDGF) and transforming growth factor beta (TGFβ) (Singer and Clark, 1999; Velnar et al., 2009). Within the tissue, monocytes differentiate to become activated macrophages. Macrophages serve integral active and regulatory roles in wound healing, and different macrophage subpopulations are present in multiple phases of wound healing. “Pro-inflammatory” M1 macrophages dominate during the inflammatory phase, while the less inflammatory M2 subtype appears later and is thought to promote tissue repair during the proliferative phase (Koh and DiPietro, 2011). During the inflammatory phase, macrophages are a critical source of cytokines such as interleukins 1 and 6, fibroblast growth factor (FGF), epidermal growth factor (EGF), TGFβ, and PDGF (Singer and Clark, 1999; Barrientos et al., 2008; Velnar et al., 2009). Signaling by these cytokines encourages the migration of keratinocytes, fibroblasts, and endothelial precursor cells to the wound bed to initiate the process of proliferative healing. Throughout the late inflammatory phase, macrophages also continue to clear debris, such as neutrophil remnants, from the wound (Velnar et al., 2009).

### Proliferation

Once the provoking injury has resolved and the initial stages of the inflammatory phase have begun (but prior to complete resolution of inflammation), the proliferative phase begins. This phase starts approximately 4 days after wounding and continues for about 2 weeks (Singer and Clark, 1999; Velnar et al., 2009). During this phase, the provisional fibrin/fibronectin ECM formed by the platelet plug is replaced with new, highly vascularized stroma. The new tissue resulting from the proliferative phase is termed granulation tissue due to the “granular” appearance endowed by its many capillaries.

Macrophages provide a continued source of numerous cytokines and growth factors to recruit and activate fibroblasts, endothelial cells, and keratinocytes to the wound and promote their differentiation (Singer and Clark, 1999; Koh and DiPietro, 2011). Fibroblasts are attracted to the wound by factors including TGFβ and PDGF from inflammatory cells and platelets (Velnar et al., 2009). These fibroblasts deposit ECM which is composed of fibronectin, collagen (primarily type III), hyaluronic acid, and proteoglycans and is less dense than normal skin ECM (Singer and Clark, 1999; Velnar et al., 2009). In the wound bed, migratory fibroblasts transition to an activated myofibroblast phenotype, marked by acquisition of alpha smooth muscle actin expression and thus contractile ability (Darby et al., 2014). These myofibroblasts begin the process of wound contraction by extending numerous pseudopods which attach to the ECM network and are then retracted to promote wound edge approximation (Velnar et al., 2009).

Wound coverage in humans is achieved via formation of a new epithelial barrier. Notably, mice, a common model in wound healing research, are loose-skinned and close their wounds predominantly by contraction (via action of the subdermal panniculus carnosus muscle). This muscle is absent in humans, where wound closure instead occurs primarily by granulation and re-epithelialization, with a much lesser degree of wound contraction (via myofibroblast contraction at the cellular level). Splinting mouse wounds to prevent contraction results in more human-like wound healing kinetics and proceeds by granulation and re-epithelialization (Galiano et al., 2004).

Complete re-epithelialization is a fundamental criterion defining successful wound healing in human patients (Pastar et al., 2014). Re-epithelialization, which begins within hours of injury (Singer and Clark, 1999), is mediated by epidermal cells and serves to re-establish the stratified squamous epithelium. Multiple populations of differentiated and stem cells contribute to this process, demonstrating remarkable cell lineage plasticity. Distinct pools of epithelial stem cells exist in the interfollicular epidermis and the hair follicle bulge, and in unwounded skin contribute to homeostasis of their respective niches. However, upon wounding, these two stem cell populations transiently lose their respective lineage identities and proliferate to help populate the new epidermis (Ge et al., 2017). Sebaceous duct cells have also been shown to dedifferentiate to repopulate the interfollicular epidermis following wounding (Donati et al., 2017).

In response to hypoxia and inflammation in the wound bed, pro-angiogenic factors (such as vascular endothelial growth factor (VEGF), FGF, and TGFβ) are released from inflammatory cells, platelets, and epidermal cells (Singer and Clark, 1999; Tonnesen et al., 2000; Johnson and Wilgus, 2014). Capillary sprouts extend from existing vessels at the wound edges and within days a new dense network of capillaries is formed in the granulating wound (Velnar et al., 2009). The high vascularity of granulation tissue encourages further recruitment of immune cells and increases oxygenation to support collagen crosslinking and wound maturation (Janis and Harrison, 2016).

### Maturation

Granulation tissue represents temporary stroma that is remodeled over time as the ECM composition shifts, the network of blood vessels is pruned, and the structure of the ECM fibrillar network undergoes organization and alignment. Granulation tissue serves as a temporary scaffold to enable fibroblasts and other cells to migrate into the wound site and guides their alignment as they deposit and organize the permanent ECM. The maturation phase is the longest phase of wound healing and may last for 1–2 years or even longer in humans (Velnar et al., 2009). The previously highly cellular tissue loses most of its cells as the remaining inflammatory cells and many fibroblasts and endothelial cells undergo apoptosis, ultimately leaving a smaller number of fibroblasts and blood vessels (Greenhalgh, 1998; Velnar et al., 2009).

Relative to the mature healed wound, granulation tissue ECM has a higher proportion of fibronectin, type III collagen, elastin, proteoglycans, and hyaluronic acid and is less dense and more hydrated. Over time fibroblasts remodel the ECM and the other structural proteins are gradually replaced to form a denser ECM composed primarily of type I collagen (Knight et al., 1993; Yates et al., 2011; Xue and Jackson, 2015). MMPs secreted by fibroblasts, keratinocytes, and resident inflammatory cells are necessary for remodeling the temporary ECM into its permanent structure (Xue and Jackson, 2015). During the proliferative phase, matrix proteins are initially laid down rapidly, in a relatively disorganized manner. In the maturation phase, the ECM molecules are reorganized and cross-linked by wound fibroblasts, thus gradually strengthening the network of fibers over time (Berthod et al., 2001; Xue and Jackson, 2015).

## Craniofacial Skin Healing

### Adult Skin Wounds Heal by Scarring

**FIGURE 2:**
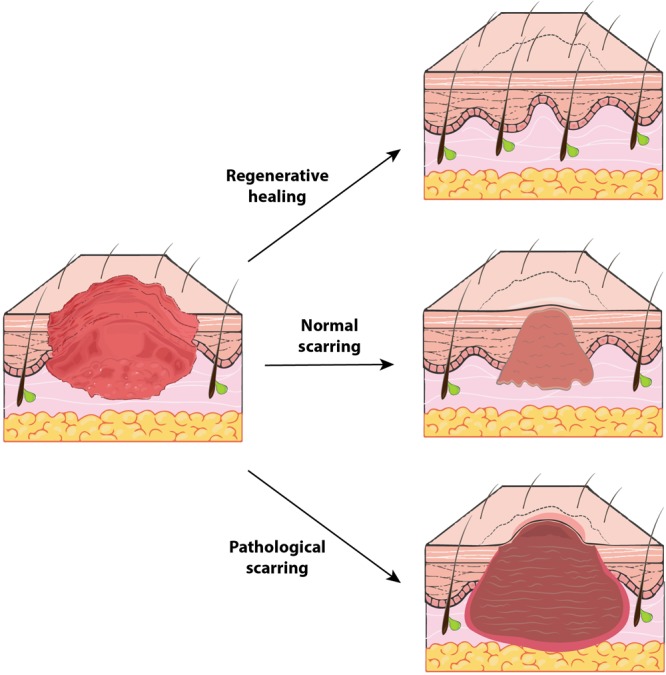
The spectrum of human wound healing. Outcomes of human soft tissue defect repair represent a broad spectrum and vary based on wound context. ***Regenerative healing*** (top) (for example, in early gestation fetuses) is the “ideal” healing outcome and results in tissue that is indistinguishable from unwounded tissue. Typical adult cutaneous healing results in some fibrosis with formation of a ***normal scar* (middle)**, which contains collagen oriented in a dense, parallel alignment and does not regrow any dermal appendages (e.g., hair follicles). In certain instances, a ***pathological scar* (bottom)**, such as a hypertrophic scar or keloid, may be generated. Compared to normal scars, these hyperfibrotic scars have even denser and more collagen, are more grossly apparent with increased discoloration, and may be pruritic or painful.

With rare exceptions, any wound to the adult human dermis will heal with some degree of scar formation (Bayat et al., 2003) (Figure 2, *middle*). Scars appear grossly as discolored and often raised areas of fibrous tissue. Scarring likely evolved as an advantageous means of rapidly limiting hemorrhage and restoring the skin barrier against environmental pathogens (Bayat et al., 2003). As such, scarring sacrifices form and function for speed in healing (and ultimately survival and procreation). Scars differ from normal, functional skin in several key ways, discussed below.

Scar tissue is permanently weaker than normal skin. Though wound strength gradually increases over the course of scar maturation, reaching 20% of its final strength during the first 3 weeks (proliferative phase) (Singer and Clark, 1999) and 50% by 6 weeks of healing (Xue and Jackson, 2015), a scar will only ever regain at most 80% of the strength of uninjured skin (Ireton et al., 2013). This is in part because scars lack rete ridges (Papel, 1992) and elastic fibers (Roten et al., 1996). Further, during the course of scar maturation, the initially disorganized collagen network undergoes alignment by myofibroblasts to form bundles of fibers that are parallel to the skin surface and to one another. This structure is less robust than the “basket-weave” pattern of unwounded skin collagen, which endows normal skin with its pliability and strength (Berthod et al., 2001). Parallel collagen organization results in scar tissue that is weak and abnormally stiff compared to healthy skin. Thus, scars are mechanically vulnerable points within the skin barrier and may shear under stress (Papel, 1992).

Adult skin wounds are also unable to regenerate melanocytes, pigment, sweat glands, or hair follicles (Singer and Clark, 1999; Hu et al., 2014; Zielins et al., 2014). Scars are therefore discolored and unable to assist in temperature regulation through sweating or piloerection (McGibbon et al., 1973; Shapiro et al., 1982). Lack of normal pigmentation and dermal appendages also contributes to unfavorable aesthetic outcomes of scarring.

Further, wound contraction driven by scar myofibroblasts is a normal, limited component of skin healing by secondary intention. However, if myofibroblasts fail to undergo apoptosis and persist in a hypertrophic scar (see Pathologic Scarring, below), painful and disfiguring contractures may form. Thus, skin scarring clearly can be both aesthetically and functionally detrimental.

### The Burden of Craniofacial Scarring

Scars may arise on the face from various forms of injury including mechanical trauma, burns, acne, and craniofacial surgery. The impact of scarring is broad: over 100 million patients per year in the developed world and many more in the developing world acquire scars following injury or surgery (Bayat et al., 2003; Bayat and McGrouther, 2005). The clinical burden of skin scars is intimately linked to their fundamental biology. As previously discussed, scar tissue is functionally inferior to healthy skin. This may be especially problematic if a large skin surface area is affected. For example, due to impaired thermoregulation, severely burned individuals are especially susceptible to developing hyperthermia and heatstroke during exercise (McGibbon et al., 1973; Shapiro et al., 1982). Furthermore, when scars cross joints they may result in contractures that limit function and, in children, interfere with limb development resulting in disfigurement and growth impairment (Goel and Shrivastava, 2010). For instance, contractures across the neck can result in mandibular deformation (Bayat et al., 2003; Goel and Shrivastava, 2010).

**FIGURE 3:**
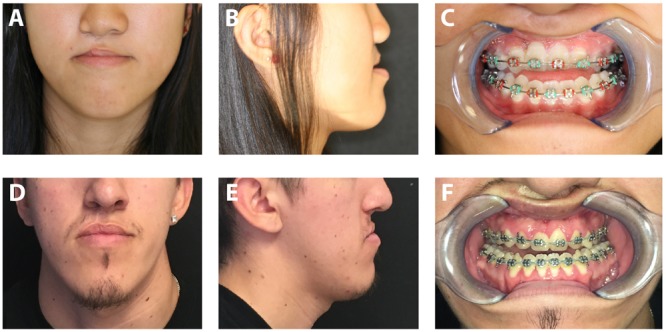
Photographs of patients with cleft lip and palate repair resulting in maxillary growth restriction. Frontal photograph **(A)** and lateral photograph **(B)** of 16-year-old female with right cleft lip and palate. Note deficient midface projection associated with maxillary hypoplasia. **(C)** Intraoral photograph of same patient demonstrating significant malocclusion. Frontal photograph **(D)** and lateral photograph **(E)** of 18-year-old male with right cleft lip and palate. **(F)** Significant malocclusion can also be appreciated in this patient on intraoral examination. *Written informed consent was obtained from the depicted individuals for the publication of these images.*

Scars around the eyes and mouth can frequently impair their opening and lead to problems with vision, speech and feeding. In the orbital region, malposition of the eyelid can be seen with entropion, ectropion, or lid retraction secondary to a cicatricial response (Ridgway et al., 2009). These deformities can result in a broad spectrum of complications with both functional and cosmetic repercussions. Excessive scarring following eyelid injury and reconstructive procedures can result in scleral show, exposure keratopathy, and symblepharon (Fogagnolo et al., 2012; Poh et al., 2014). In the midface, scar formation following cleft lip and palate repair can impact both soft tissue function and bony growth. Despite palatoplasty, scarring of the soft palate can still result in velopharyngeal dysfunction in 20–30% of patients, requiring prosthetic management or surgical revision to improve speech (Pai et al., 2018). Long-term sequelae of midface scarring on facial growth can also be noted in many patients following cleft repair, with three-dimensional growth retardation in the maxilla resulting in malocclusion which may be difficult to correct with orthodontics alone (Figure 3). Techniques to limit palatal scar contraction have thus been sought to minimize subsequent maxillary growth restriction (Levi et al., 2009).

In the skin, pathological scars such as hypertrophic scars and keloids can be unsightly, itchy, and painful, and sometimes require surgical revision to alleviate symptoms. Unfortunately, these surgeries represent a second injury that may heal with repeated pathological scar in certain individuals. The burden of dysfunction and disfigurement associated with scars can also negatively impact psychological health, particularly of children (Gibson et al., 2018). This is particularly true of craniofacial scars, which have been linked by several studies to an especially high risk of depression and lower quality of life (Rumsey and Harcourt, 2004; Roberts and Gierasch, 2013). Considering their broad clinical implications, it is not surprising that scars are responsible for over $20 billion in annual healthcare expenditures in the United States (Sen et al., 2009).

### Factors Influencing Scarring

Several factors influence the process of scarring. The first is wound etiology, as it pertains to the depth of injury, degree of tissue destruction, and introduction of pathogens. For scarring to occur, injury must involve the dermis, and pathologic forms of wound healing (see below) are more likely to occur if injury involves the bottom one-third of the dermis or is associated with infection (Huang et al., 2014; Ogawa, 2017). The second factor is the location of the wound, due to variations in mechanical forces across the skin at different anatomical sites. On the face, tension lines arise from interactions between the skin and the underlying muscles of facial expression (Son and Harijan, 2014). Wounds running across a tension line experience greater perpendicular force and must respond with greater collagen deposition to hold the skin together, resulting in a larger scar (Son and Harijan, 2014). Craniofacial surgeons have long exploited this property of the skin by incising along relaxed skin tension lines and aiming to reduce traction on suture lines, thus facilitating wound healing with less scarring. The third factor is patient demographics, as it has been observed that darker-skinned and younger individuals are at higher risk to heal pathologically with hypertrophic scars and keloids, though the causative mechanisms explaining these risks are not well-understood (Gauglitz et al., 2011).

### Pathologic Scarring: Hypertrophic Scars and Keloids

Hypertrophic scars and keloids result when wounds heal with excessive fibrosis (Figure 2, *bottom*). Hyperproliferative scarring is for the most part unique to humans among mammals. Such pathological scars are frequently encountered in the craniofacial region following burns, lacerations, piercings, infections, and surgical procedures such as head and neck cancer resection, in which tissue injury occurs deeper in the reticular dermis (Huang et al., 2014; Ogawa, 2017). In contrast to “normal” scars that are flat and relatively pliable, hypertrophic scars are raised, stiff, and may be pruritic or painful. As alluded to above, tension is a contributing factor to hypertrophic scarring and is putatively sensed and transduced by myofibroblasts into increased proliferation and collagen deposition (Son and Harijan, 2014). Accordingly, hypertrophic scars often develop on areas of the body with the thickest dermis and greatest skin tension (e.g., back, chest, upper arms). Hypertrophic scars in the craniofacial region are often a result of burn injuries, which demonstrate a predilection for pathological healing (Deitch et al., 1983; Finnerty et al., 2016).

While scars and hypertrophic scars remain confined to the wound margins and typically regress with time, keloid scars may extend far beyond the wound margins, grow continuously, and often recur after excision. Molecular studies of hypertrophic scar and keloid fibroblasts show chronic inflammation (e.g., interleukins 6 and 1B, tumor necrosis factor alpha) and loss of p53 expression in the latter, supporting the notion that keloids are actually benign tumors in which the proliferative phase of healing continues indefinitely (Ladin et al., 1998; Ogawa, 2017). Thus, it has been proposed that normal scars, hypertrophic scars, and keloids lie on a spectrum of increasing fibrotic response to injury (Huang et al., 2014). Indeed, it can be difficult to distinguish these fibroses clinically and one injury may produce regions of both normal and abnormal scarring that regress or progress over time. Though there are some histological features often found in keloid scars (e.g., thick bundles and whorls of collagen), these same fibrous features may be seen in severe hypertrophic scars. Thus, the clinical evaluation of fibroses is a qualitative exercise based in “gestalt” or subjective scoring. It remains to be seen whether scars can be clinically evaluated using quantitative metrics of the underlying connective tissue architecture.

## Wound Healing of the Oral Mucosa

**FIGURE 4:**
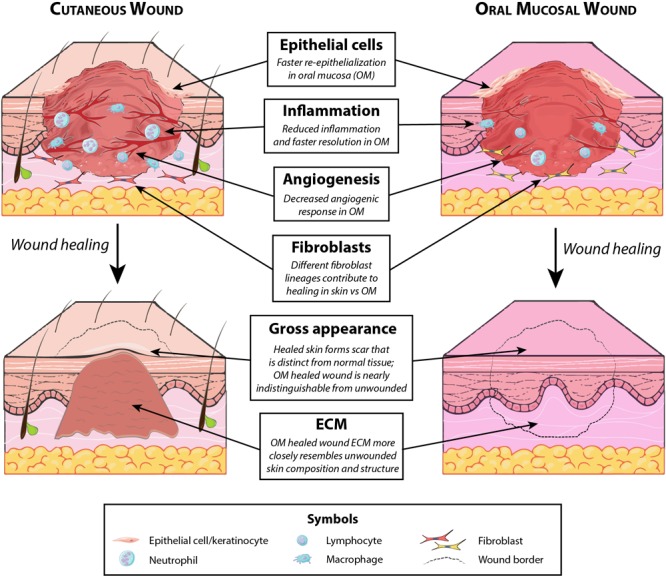
Key differences between cutaneous and oral mucosal wound healing. Several distinctions exist between typical healing of cutaneous wounds involving the dermis (which results in fibrosis) and uncomplicated oral mucosal healing (which is minimally scarring). Notable differences, summarized above, exist both in the healing wound (with regard to participating cells and degree of angiogenesis) and in the fully healed wound (with regard to resulting matrix composition and gross appearance).

While injuries to the dermis of the head and neck heal by scarring, a markedly different phenotype is observed in the oral cavity. Wounds to the oral mucosa mostly heal with minimal to no scar, by regenerating tissue that is largely indistinguishable from unwounded oral mucosa (Wong et al., 2009) (Figure 2, *top*). Oral mucosal wounds also exhibit accelerated healing with rapid re-epithelialization compared to skin wounds (Johnson et al., 2014; Iglesias-Bartolome et al., 2018). While oral mucosal wound repair has been studied to a lesser extent than cutaneous healing, several key principles governing wound healing in the oral cavity have been elucidated and are reviewed in the following sections. The differences between cutaneous and oral mucosal healing are summarized in Figure 4.

### Role of Environment in Scarless Oral Healing

Although wound healing in the oral cavity proceeds along the same fundamental pathways as wound repair at other anatomic sites, there are notable intrinsic and extrinsic factors that differ between dermal and oral mucosal tissue repair (Larjava, 2012). Many studies have suggested that the unique environment of the mouth plays a critical role in regeneration. In addition to the moisture and temperature of the oral cavity, the presence of saliva represents a distinguishing feature of the intraoral environment and has been identified as a key factor in the accelerated regeneration of the palate and gingiva (Szpaderska et al., 2003; Glim et al., 2013). Saliva supports wound repair by assisting oral fibroblasts in wound closure, increasing cell turnover and stimulating the release of growth factors in order to achieve rapid oral wound healing. Salivary gland secretions also contain EGF that may facilitate wound healing (Noguchi et al., 1991), which has been proposed as the reason for animal behaviors such as wound licking. Human saliva also contains histatins, peptides with antimicrobial properties that promote fibroblast and keratinocyte migration, further enhancing the minimally scarring wound healing response in the oral cavity (Boink et al., 2016).

### Reduced Inflammation in Oral Healing

As discussed previously, the response to tissue injury begins with hemostasis followed by recruitment of cytokines. These events trigger an inflammatory response which is thought to be critical in the initiation and modulation of scar formation. Persistent inflammation is a hallmark of chronic wounds and impairs tissue repair, delaying healing and increasing fibrosis, whereas reduced inflammation is associated with a less severe fibrotic response (Eming et al., 2007; Larjava, 2012). Previous studies have demonstrated that the inflammatory response during oral wound repair is markedly attenuated and resolves more quickly when compared to that of cutaneous wound healing (Mak et al., 2009; Iglesias-Bartolome et al., 2018). Levels of pro-inflammatory cytokines (e.g., interleukin 6) and inflammatory cells (e.g., neutrophils, macrophages, T-cells) are reduced both within the oral mucosa at baseline (Glim et al., 2015) and within oral wounds (Szpaderska et al., 2003). Oral mucosal epithelial cells also exhibit a diminished response to inflammatory stimuli *in vitro* compared to skin epithelial cells, suggesting that in addition to the reduced inflammation seen in oral healing, oral epithelial cells are also intrinsically less reactive to inflammation (Chen et al., 2010). The attenuated oral mucosal inflammatory response may allow for faster and less fibrotic wound healing.

### Differences in Angiogenesis

Interestingly, compared to skin, the oral mucosa displays decreased levels of VEGF and a more muted angiogenic response to wounding (Szpaderska et al., 2005). This finding may be related to the less severe tissue hypoxia experienced by oral wounds (Chen et al., 2012). The finding of decreased angiogenesis in the setting of oral mucosal healing does not have immediately clear implications and is particularly curious given that unwounded oral mucosa is more highly vascularized than skin (Glim et al., 2015). Further research is needed to determine the therapeutic relevance of these observations.

### Complicated Wound Healing in the Oral Cavity

As discussed above, wound repair in the oral cavity (specifically, of wounds involving the palatal mucosa only and gingiva) is generally characterized by healing with minimal scar formation. However, a more complex repair process can be seen in other areas of the oral cavity or when healing is complicated by additional factors, and may lead to compromised wound healing outcomes. Similar to disordered tissue repair in the skin, complicated wound healing in the mouth may result in poor clinical outcomes such as fistulas, sinus polyps, wound necrosis and dehiscence, or, at the other end of the spectrum, excessive fibrosis (Guo and Dipietro, 2010; Glim et al., 2013; Politis et al., 2016).

Complicated wound healing can occur after dental surgery, posing a significant burden on patients undergoing intensive dental procedures. This phenomenon can be observed in periodontal healing following tooth extraction as it involves repair of not only the oral mucosa, but the remainder of the periodontium including the alveolar bone, cementum, and gingiva. Although gingival repair mimics typical wound healing in the oral mucosa, ultimately healing with little to no fibrosis, the bone healing and remodeling process in periodontal tissue continues for up to 6 months and may be associated with resorption of the alveoli, bone loss, and inflammation (Guo and Dipietro, 2010; Glim et al., 2013; Politis et al., 2016).

Another example of complicated intraoral healing occurs within the dental pulp. Exposure of the dental pulp may cause superficial necrosis and trigger an inflammatory response. This inflammatory response leads to the recruitment of dental pulp progenitor cells, which differentiate to produce dentin in order to repair the exposed pulp. However, prolonged inflammation in this area is likely to lead to chronic necrosis (Goldberg, 2011; Larjava, 2012; Politis et al., 2016). In addition to pulp necrosis, disadvantageous outcomes such as loss of alveolar bone, breakdown of dental papilla, and unstable bony fragments are potential complications of pulp healing in the setting of unresolved inflammation (Larjava, 2012).

Finally, although wound healing in the oral cavity does not classically result in a scar response, severe fibrosis can occur following palatal healing when healthy bone is not present underlying the wound (Politis et al., 2016). Harvesting mucosa and underlying buccinator muscle as part of a facial artery musculomucosal flap has also been associated with excessive scarring and trismus in 2% of patients (Ayad and Xie, 2015), which may require additional surgical release. Similar to scarring of the skin, fibrosis within the mouth can cause a host of functional limitations; a common example is presented in the following section.

## Clinical Correlate: Cleft Lip and Palate

Cleft lip and/or palate is one of the most common congenital defects, with cleft lip present in an estimated 1 in 940 live births and cleft lip and/or palate collectively affecting over 7,000 infants annually in the United States (Parker et al., 2010). While numerous contributing risk factors have been identified, the precise causes of cleft lip and palate remain unknown. Treatment involves complex surgical repair of the cleft. The goals of care include intact primary and secondary palate; normalized aesthetic appearance, hearing, and speech; and normal psychosocial development (Denadai and Raposo-Amaral, 2018). Lip repair is typically done at 2–3 months of age, and palate repair is performed between 6 and 12 months (Nahai et al., 2005).

Due to the skin wounds involved in these surgeries and the underlying bony defect in the case of cleft palate, healing of cleft lip and palate repair results in fibrotic (scarring) outcomes for both the lip and the palate. Scarring in this situation can have an array of aesthetic and functional consequences for these young patients throughout their development. Hypertrophic scarring can be common following cleft lip repair – reported incidence rates of post-operative hypertrophic scarring vary but average around 25%, with rates varying by ethnicity and as high as 36.3% in Asian patients (Soltani et al., 2012; Papathanasiou et al., 2017). As previously discussed, hypertrophic scars can be itchy and painful and are associated with increased functional and cosmetic disability from scarring (Rabello et al., 2014). Scarring from cleft lip repair can cause lip asymmetry as the scar contracts leading to a shortened lip and nasal deformity on the affected side (Soltani et al., 2012). Such scarring may require further surgical revision to restore normal aesthetics and function, which causes psychological stress, risk associated with additional surgeries and anesthesia exposure, and significantly increased cost of treatment (Sitzman et al., 2015).

Cleft lip and palate repair and revisions can also lead to serious functional detriment. Maxillary hypoplasia, or underdevelopment of the maxillary bone leading to a reduction in its dimensions along all axes, is the most common secondary deformity as a result of cleft lip/palate and their initial repair (Richardson et al., 2018) (Figure 3). Maxillary hypoplasia is thought to be due to a combination of intrinsic primary growth deficiency of the maxilla and fibrosis of the lip and palate resulting from surgery (Oberoi et al., 2012; Richardson et al., 2018). Specifically, the formation of stiff, fibrotic scar tissue following the primary repair operation is believed to impair maxillary blood supply and to physically tether the developing maxilla, restricting its anterior and lateral growth (James and Brook, 1985). Thus, scarring is directly implicated in the pathogenesis of maxillary hypoplasia. This growth impairment and the resulting constraint on soft palate movement can cause an abnormal profile as well as velopharyngeal dysfunction affecting speech (“nasal escape”), and may require further surgeries to repair (James and Brook, 1985; Oberoi et al., 2012; Woo, 2012). The reported prevalence of maxillary hypoplasia requiring surgical correction is between 14 and 45% (Oberoi et al., 2012). Furthermore, scarring of palatal tissues can also impact outcomes of secondary procedures such as closure of oronasal fistulas, with recurrence rates as high as 25% thought to be due to scarcity of healthy pliable tissue and tension on the suture line (Sahoo et al., 2016). The frequency of scarring-related complications in this pediatric patient population, especially considering the highly visible and functionally critical site of scarring, highlights the need for novel treatments to target scarring.

## Current Therapies for Scarring

Many different therapeutic approaches have been developed to minimize the appearance and functional impact of scars. Therapies delivered at the time of wounding include dressings, tapes, and silicone sheets designed to reduce tension on the wound and suture lines (Atkinson et al., 2005; Chen and Davidson, 2005; O’Brien and Pandit, 2006; Thomas and Somenek, 2012). For example, the embrace device is an adhesive silicone tension-offloading device that was shown in a randomized clinical trial to significantly improve the appearance of suture line scars in patients undergoing elective abdominoplasty (Longaker et al., 2014). Injected therapies delivered over the course of healing include corticosteroid injections to minimize inflammation (Chowdri et al., 1999), chemotherapeutic agents such as 5-fluorouracil to prevent excessive fibroblast proliferation (Uppal et al., 2001), and botulinum toxin (Ziade et al., 2013). Unfortunately, these injected agents have shown varying clinical efficacy and their therapeutic effects are often short-lived. Another class of therapies is delivered after formation and maturation of the scar. For example, pulsed dye lasers and cryotherapy with liquid nitrogen have been shown to improve scar appearance by thermally inducing coagulative necrosis of wound blood vessels (Layton et al., 1994; Kuo et al., 2004; Nouri et al., 2010). Radiation has also been employed after resection of keloids to minimize recurrence, with some success (Sclafani et al., 1996). Finally, if the scarred area is refractory to treatment and sufficiently matured, surgical revision may be pursued. This process entails resecting the scar and repairing the skin in alignment with tension lines, which may require re-orientation (Thomas and Somenek, 2012). The scar revision surgery itself results in a secondary scar which may require further treatment; tension shielding using the embrace device has been shown to effectively decrease scarring following scar revision surgery (Lim et al., 2014).

Regarding the currently available therapies for scar management, we identify two major limitations. First, there is no quantitative basis on which to objectively judge the effects of vulnerary agents beyond visual analog scores or connective tissue histology. Outcomes are therefore subject to inter- and intra-observer biases. Future approaches should be validated against quantitative benchmarks of wound healing. Second, although some approaches have been shown to improve scar appearance, to date there is no available therapy that supports true regeneration of skin with secondary dermal elements. Agents targeting the TGFβ signaling pathway and various cellular treatments using autologous adipose- and bone marrow-derived stem cells showed promise in preclinical trials but have not demonstrated clinical efficacy (So et al., 2011; Little et al., 2012). Considering the physiologic and pathologic role that mechanical forces play in wound healing and scar remodeling (Akaishi et al., 2008; Ogawa et al., 2012; Huang et al., 2017). and given the observation that tension offloading reduces scarring (Lim et al., 2014; Longaker et al., 2014), we believe agents targeting mechanotransduction signaling may yield a more regenerative phenotype. For example, Wong et al. (2011) demonstrated that scar fibrosis is dependent on stiffness signaling through focal adhesion kinase (FAK) – thus, the cellular stiffness sensing apparatus (FAK, ROCK) and its downstream transcriptional effectors (YAP/TAZ) may represent a viable therapeutic target to promote skin regeneration.

## Future Directions: Toward Scarless Craniofacial Healing

The lack of current effective treatments for preventing scarring and promoting regeneration in wound healing highlights the necessity of continued research. Regenerative healing is a rare phenomenon in adult mammals, with oral mucosa, skeletal muscle, and liver representing the few known examples of complete or partial adult regeneration capacity (Michalopoulos and DeFrances, 1997; Stoick-Cooper et al., 2007). Many factors have been elucidated as distinguishing scarless versus scarring healing in, for example, the oral mucosa compared to the dermis (see Wound Healing of the Oral Mucosa, above). However, it is incompletely understood how these different factors contribute to scarless versus scarring healing, and discoveries regarding the basis of scarless healing in the oral cavity have yet to be translated to therapeutics for targeting skin scarring. Elucidating the cellular and molecular mechanisms behind physiologic examples of regenerative healing (such as oral mucosal healing and early gestation fetal healing) may provide valuable insights that can be applied to developing novel therapies.

### Fibroblast Heterogeneity and Scarring

Fibroblasts are central to both the production and remodeling of scar tissue. While fibroblasts were long believed to be a fairly homogeneous cell population, recent work has shed light on the striking heterogeneity of fibroblasts within the dermis and throughout the body (desJardins-Park et al., 2018). Indeed, fibroblast heterogeneity is a burgeoning field and may represent a lens through which the differences in skin and oral cavity healing can be better understood. Specific fibroblast subpopulations contribute differentially to wound healing at various sites in the body (Driskell et al., 2013; Rinkevich et al., 2015; desJardins-Park et al., 2018). In 2015, our laboratory demonstrated that embryonic *En1* expression marks a distinct lineage of fibroblasts responsible for the majority of scarring and wound healing on the dorsum of mice (Rinkevich et al., 2015). In the oral cavity, *Wnt1* expression was found to demarcate a fibroblast lineage that contributes to oral mucosal healing (Rinkevich et al., 2015). Critically, transplantation of *Wnt1* lineage-positive fibroblasts derived from the oral cavity into the dorsal skin yielded minimal fibrosis, whereas *En1* lineage-positive fibroblasts from the dorsal dermis transplanted into the oral mucosa exhibited a scarring phenotype (Rinkevich et al., 2015), demonstrating that adult dermal fibroblasts possess intrinsic scarring ability whereas oral mucosal fibroblasts are intrinsically less fibrotic.

The resident gingival fibroblasts that accomplish wound healing have also been shown to be neural crest-derived (*Wnt1* is a neural crest marker) (Rinkevich et al., 2015; Isaac et al., 2018). Consistent with their developmental origin (Ransom et al., 2018), these oral mucosal fibroblasts also demonstrated multipotential differentiation capacity (Isaac et al., 2018), which is significant in light of their ability to contribute to tissue regeneration. Studies have also demonstrated that fibroblasts in the oral cavity have an extended replicative capacity and are less susceptible to differentiation into myofibroblasts, thus preserving their non-scarring phenotype (Enoch et al., 2007; Meran et al., 2007). Interestingly, another study demonstrated greater numbers of myofibroblasts, but decreased wound contraction, in oral wounds compared to skin wounds (Mak et al., 2009), highlighting the importance of varying fibroblast phenotypes and not merely cell numbers. These findings suggest that the regenerative wound healing phenotype observed in the oral cavity and the scarring phenotype of the dermis are at least in part modulated by properties intrinsic to their resident fibroblasts.

### Molecular Signature of Wound Healing

Recent work has shed increased light on the molecular differences between oral and skin wound healing. Oral wounds have been found in several studies to have decreased expression of TGFβ, specifically the pro-fibrotic TGFβ1 isoform, compared to cutaneous wounds (Szpaderska et al., 2003; Schrementi et al., 2008; Mak et al., 2009). More subtly, multiple studies have suggested that the oral mucosa exists in a state that is transcriptionally “primed” for wound repair. One such study demonstrated that fewer gene changes occur upon wounding in the oral mucosa compared to skin (Chen et al., 2010). Combined with the fact that the oral mucosa exhibits relatively high constitutive expression of factors important to tissue repair (e.g., growth factors, cytokines, elements of host defense) (Warburton et al., 2005), it appears that the oral mucosa is unusually “ready” to respond to tissue injury. A recent study also found that the same transcriptional networks that are activated upon wounding are also present at baseline in the oral mucosa (Iglesias-Bartolome et al., 2018). These findings are consistent with the fact that the oral mucosa likely undergoes frequent minor trauma (e.g., cheek biting, aphthous ulcers), and may therefore have undergone adaptation to prioritize more rapid wound healing. Moving forward, it will be interesting to examine whether such molecular profiling may reveal insights into specific mediators of fibrosis versus regeneration.

### Epithelial Cell Differences

Due to the highly accelerated re-epithelialization that occurs in oral mucosal wound healing, differences between oral and cutaneous epithelial cells have been the subject of much research. Studies have disagreed on whether a significant difference exists in proliferation between oral and cutaneous keratinocytes (Glim et al., 2014; Iglesias-Bartolome et al., 2018), possibly attributable to differences in analysis methods. However, multiple studies found that oral keratinocytes exhibit decreased differentiation compared to skin keratinocytes (Glim et al., 2014; Iglesias-Bartolome et al., 2018), potentially contributing to regenerative capabilities. The larger supply of less-differentiated cells found in the oral mucosa at baseline could also contribute to its ability to rapidly respond to injury and achieve wound closure. Recent work has further defined this pool of “regenerative” epithelial cells, demonstrating that long-lived oral epithelial progenitor cells (which express Bmi1) are found throughout the basal layer of the oral mucosa, and rapidly divide to give rise to daughter cells which begin the process of differentiation while in the basal layer (Jones et al., 2018).

### Models of Scarless Healing

In early gestation mammalian fetuses, cutaneous wounds heal by complete regeneration, with no scar formation (though other tissue insults such as gastric and intestinal damage heal with scar) (Larson et al., 2010) (Figure 2, *top*). Fetal skin healing represents another important paradigm for understanding regenerative healing. Interestingly, oral mucosal wound healing recapitulates several key distinguishing features of fetal wound healing. Fetal wounds, similar to wounds of the oral mucosa, exhibit minimal inflammation (Larson et al., 2010). Further, healed wound ECM in the oral mucosa has features resembling fetal skin and wound ECM such as higher fibronectin content, and both fetal and oral wounds exhibit increased vascularity compared to typical adult cutaneous wounds (Glim et al., 2014).

Models of scarless wound healing in adult mammals remain scarce but sought-after, as examples of postnatal mammalian skin regeneration may yield particularly relevant insights for patient treatment. A recent study by Golberg et al. induced skin damage in rats using irreversible electroporation, provoking widespread cell death but preserving ECM architecture. The ablated tissue was observed to undergo regeneration (including regrowth of hair follicles and sebaceous glands), posing a potential new model for studying scarless healing and suggesting a critical role of the ECM in orchestrating wound healing (Golberg et al., 2018). Another intriguing model of scarless healing is the African spiny mouse (*Acomys* spp.), which possesses a unique capacity for complete skin regeneration following cutaneous wounding (Seifert et al., 2012). Similar to fetal and oral mucosal healing, *Acomys* wounds exhibit a significantly muted inflammatory response, as well as a decreased MMP/TIMP ratio (Maden and Brant, 2018). Recent work has also found differences in *Acomys* hair follicles, which have a larger bulge area and increased expression of stem cell markers (K15, CD34, and Sox2) (Jiang et al., 2019); and dermal fibroblasts, which exhibit decreased myofibroblast activation in response to substrate stiffness (Stewart et al., 2018).

### Toward Novel Therapeutics

While scar treatment options remain limited, recent basic science discoveries continue to pave the way for clinical advancement. Biomaterials science represents a rapidly growing field working toward developing innovative dressings and materials for wound treatment and scar prevention. For example, Qi et al. reported that hydrogels made from sericin (a silk-derived natural biomaterial) resulted in accelerated healing with reduced inflammation and features of regeneration (e.g., increased hair follicle neogenesis) in a preclinical mouse model (Qi et al., 2018). Identification of novel anti-scarring drugs is also an active area of research; for instance, a study from our laboratory recently established doxycycline as a putative anti-scarring agent which reduces fibrosis and promotes features of regeneration in a splinted mouse wound healing model (Moore et al., 2018). Translation of these discoveries into a clinical setting, and continued exploration of scarring from the lenses described in this article, will lead to continued advancement in our ability to treat and improve craniofacial wound healing.

## Conclusion

Scarring and fibrosis represent a massive burden of disease both in the United States and worldwide. Skin scarring is not only an aesthetic problem but often a deeply functional one, particularly in the setting of craniofacial wound healing and in the pediatric patient population. Wound repair outcomes differ markedly between different sites, with oral mucosal healing, which typically results in minimal scarring, representing a dramatic exception to the fibrotic healing outcomes seen in most other organs including the skin. Recent research has revealed numerous insights into differences in inflammation, fibroblast populations, and keratinocytes that may contribute to regenerative healing in the oral cavity. Continued exploration into mechanisms of scarless and scarring wound healing will drive the development of novel anti-scarring therapies which have the potential to improve the lives of millions of patients.

## Author Contributions

HJ-P, SM, MSC, DW, and ML wrote the manuscript. HJ-P, SM, and DW created the figures. HJ-P, DW, and ML edited the manuscript.

## Conflict of Interest Statement

ML is co-founder of, has an equity position in, and is on the Board of Directors of Neodyne Biosciences, Inc., which developed the embrace device. The remaining authors declare that the research was conducted in the absence of any commercial or financial relationships that could be construed as a potential conflict of interest.
